# Comparison of serum levels of interleukin 33 in combination with serum levels of C-reactive protein, Immunoglobulin G, Immunoglobulin A, and Immunoglobulin M in recurrent pregnancy loss: A case-control study

**DOI:** 10.18502/ijrm.v22i4.16392

**Published:** 2024-06-12

**Authors:** Hossein Ali Khazaei, Farahnaz Farzaneh, Saeedeh Sarhadi, Javid Dehghan Haghighi, Forough Forghani, Vahid Sheikhi, Bahman Khazaei, Lida Asadollahi

**Affiliations:** ^1^Clinical Immunology Research Center, Zahedan University of Medical Sciences, Zahedan, Iran.; ^2^Department of Obstetrics and Gynecology, School of Medicine, Iran University of Medical Sciences, Tehran, Iran.; ^3^Health Promotion Research Center, Zahedan University of Medical Sciences, Zahedan, Iran.; ^4^Department of Community Medicine, School of Medicine, Zahedan University of Medical Sciences, Zahedan, Iran.; ^5^Department of Obstetrics and Gynecology, School of Medicine, Zahedan University of Medical Sciences, Zahedan, Iran.; ^6^Department of Pediatrics, School of Medicine, Zahedan University of Medical Sciences, Zahedan, Iran.; ^7^School of Medicine, Zahedan University of Medical Sciences, Zahedan, Iran.

**Keywords:** Interleukin-33, Abortion, IgG, IgA, IgM.

## Abstract

**Background:**

One of the critical cases of recurrent pregnancy loss is immunological factors, whereas obtaining effective prevention or treatment is necessary for cognition of reasons.

**Objective:**

In this study, we tried to evaluate some immunological factors related to recurrent pregnancy loss.

**Materials and Methods:**

This case-control study was conducted on 66 women at the age of 18–35 yr who were referred to the Clinic of Gynecology and Obstetrics, Ali Ibn Abi Taleb hospital, Zahedan, Iran, from August-December 2019. Interleukin 33 (IL-33) serum levels were measured using enzyme-linked immunosorbent assay. Immunoglobulin G, Immunoglobulin A, Immunoglobulin M (IgM), and C-reactive protein levels were measured by serology and hematology methods.

**Results:**

The mean age of total participants was 30.8 ± 3.80 yr. The mean serum IL-33 in the case group was 318.5 ± 254.1 pg/ml and was lower than the control group (354.2 ± 259.9 pg/ml), which was not statistically significant (p = 0.52). The level of C-reactive protein in the case and control was not significantly different (p = 0.27), and Immunoglobulin A and Immunoglobulin G in the case and control were also not significantly different (p = 0.46, and p = 0.16, respectively), but there were significant differences (p = 0.003) between the level of the IgM in the case and control groups.

**Conclusion:**

No statistically significant difference was observed in the IL-33 serum level, for at least 4–6 months after the last abortion in the case group and the final live birth in the control group. In contrast, serum levels of IgM were statistically significant. Finally, the need for more studies is felt according to the different results of the previous studies in this field.

## 1. Introduction

About 1–2% of women trying to get pregnant experience recurrent pregnancy loss (RPL) (1, 2). For the medical and scientific communities, RPL is challenging. In reality, only about 50% of RPL cases can be defined (3). The causes of RPL are chromosomal defects of the fetus, uterine disorders, infections, hormonal or endocrine causes, disorders of immunological factors, hereditary and acquired thrombophilia, or environmental and nutritional factors. However, in more than 50% of cases, the reasons for abortion are etiologically unknown (2). Studies have shown that chromosomal abnormalities in miscarriage in the first trimester are well established and that about 5% of couples with 2 or more miscarriages carry chromosomal structural abnormalities or may have multiple problems simultaneously (4–6). The innate and adaptive immune system plays a significant role in endometrial remodeling and maternal tolerance towards the embryo (7).

On the other hand, a successful pregnancy depends on the role of inflammatory markers, as mentioned above, which can be protective or harmful to fertilization. It has been suggested that a balance between pro-inflammatory and anti-inflammatory factors such as C-reactive protein (CRP), Immunoglobulin G (IgG), Immunoglobulin A (IgA), Immunoglobulin M (IgM), and cytokines is essential for successful pregnancy (8, 9). Interleukin (IL) 33, which is a member of the IL-1, plays an important role in host defense, immune regulation, neuronal damage, and inflammation (10, 11). Most of the markers reported in previous studies showed that the role of regulatory T cells in RPL in Iranian women is highlighted by their association with them. Endothelial, epithelial, T helper 2-activated, and mast cells are the main cells that express IL-33 (9, 12). Endothelial cells and smooth muscle cells within the placenta, chorioamniotic membranes, and umbilical cord are where the expression of IL-33 occurs. Also, some studies have shown that IL-33 serum levels in recurrent miscarriages are different compared to normal pregnancies (10, 13). Another study reported that IL-33 serum levels were significantly lower in women with recurrent miscarriages than in control group (14). It also shows what cellular and molecular mechanisms may be involved in the dysregulation of IL-33 signaling and poor pregnancy outcomes in women (11). This complexity further fuels the ongoing controversy about which immunological factors play a role in the pathogenesis of RPL (15).

Due to no information available and the role of immune function, we aimed to evaluate and compare the IL-33 serum level some related immunological factors in patients with RPL and normal individuals referred to the gynecology and obstetrics clinic of Ali Ibn Abi Taleb hospital in Zahedan, Iran.

This study aimed to determine the association of the serum levels of IL-33, CRP, IgG, IgM, IgA with RPL.

## 2. Materials and Methods

This case-control study was conducted on 66 women aged between 18 and 35 yr, who referred to the Clinic of Gynecology and Obstetrics, Ali Ibn Abi Taleb hospital, Zahedan, Iran from August-December 2019. Participants were selected by an easy and accessible method and divided into 2 groups of 33 as a case (women with a history of RPL) and 33 as the control group (including healthy women).

Inclusion criteria were having a history of recurrent miscarriage (3 consecutive miscarriages before 20 ${\;}^{\text{th}}$ wk), at least 4–6 months after the last miscarriage.

The control group included 33 healthy women aged between 18 and 35 yr without a history of abortion and who have at least one child (at least 4–6 months have passed since the last delivery).

Those women having uterine anatomical disorders, such as double uterus, arcuate uterus, unicornuate uterus, bicornuate uterus, septate uterus, thyroid disorders, systemic diseases such as antiphospholipid syndrome, systemic lupus erythematosus, rheumatoid arthritis, hyperprolactinemia, chromosomal problems of parents, and allergic diseases were excluded from the study. 6 women were excluded according to exclusion criteria (autoimmune diseases, history of allergic reactions, hypothyroidism).

In this study, repeated abortions meant losing pregnancy products before the 20 ${\;}^{\text{th}}$ wk of pregnancy on at least 3 consecutive or non-consecutive occasions. 5 cc of peripheral blood was taken from each participant to determine the serum levels of IL-33, CRP, IgG, IgA, and IgM and sent to the laboratory of Ali Ibn Abi Taleb hospital, Zahedan, Iran. The blood samples were centrifuged according to the kit instructions. The serum was separated and stored in the laboratory freezer until the end of the study. Finally, after completing the number of samples, the IL-33 serum level in 2 groups was measured by enzyme-linked immunosorbent assay method (EASTBIOPHARM CO., China).

### Sample size

A preliminary estimate of the study's sample size was calculated from a previous study (16). Mean IL-33 serum levels were used, with a type I error of 0.05 and a type II error of 0.2. According to the comparison of the 2 mean formulas, each group necessarily had at least 12 participants. During the study, this number increased to 33 patients in each group.

### Ethical considerations

This study was approved by the Ethical Committee of Zahedan University of Medical Sciences, Zahedan, Iran (Code: IR.ZAUMS.REC.1398.217). Participants were enrolled after obtaining informed written consent.

### Statistical analysis

The Kolmogorov-Smirnov test was used to evaluate the normal distribution of data. Due to the existence of abnormal distribution in quantitative variables, Mann-Whitney non-parametric test was used to compare the level of CRP and antibodies between case and control groups. P < 0.05 was considered significant. IBM SPSS Statistics for Windows, version 22.0 (IBM Corp), was used for the analysis, 2013, IBM Corp., Armonk, New York.

## 3. Results

In this study, 66 women were divided into 2 groups of 33, a case group (women with a history of RPL) and 33 as a control group (including healthy women) referred to the clinic of gynecology and obstetrics, the mean age of the participants was 30.8 ± 3.80 with a maximum and minimum value of 19.35 respectively. The mean age of participants in the group with recurrent abortion (case) was 30.9 ± 3.1 yr, and for women without recurrent abortion (control) was 30.7 ± 4.4 yr (p = 0.73). Those who met the study inclusion and exclusion criteria were investigated. It should be noted that due to the lack of normal distribution of 2 variables, age (p < 0.001) and IL-33 (p = 0.01), the nonparametric test was used to compare the 2 groups. It is also indicated that the mean age of participants in this study was 30.8, with a standard deviation of 3.8. No significant difference was observed in age distribution between the case and control groups (p = 0.73).

The mean and standard deviation of IL-33 serum levels in the case and control groups are shown in table I. According to the results of this study, the mean in the group of patients with recurrent miscarriage was 318.5 pg/ml with a standard deviation of 254.1, and the mean serum level of IL-33 in the group of healthy women without recurrent miscarriage was obtained as 354.2 pg/ml with standard deviation 9/255. According to table I, the mean serum level of IL-33 in the case group (318.5 pg/ml) was lower than the mean in the control group (354.22 pg/ml). Still, this difference was not statistically significant (p = 0.52, Table I).

Furthermore, the results of this study showed that CRP levels were not significantly different in case and control groups (p = 0.27). Regarding IgA and IgG, no significant difference was observed in the levels of these antibodies in the 2 groups with and without a history of recurrent miscarriage (p = 0.16, p = 0.46). While the difference in IgM level between the 2 groups was statistically significant (p < 0.001). The observed serologic patterns are detailed in table II.

**Table 1:** Comparison of the mean and standard deviation of IL-33 serum levels in case and control groups


				**95% CI**	
**Groups**	**IL-33 (pg/ml)**	**P-value**	**Mean difference**	**Lower**	**Upper**
				<statement> <title>Case </title> </statement>	318.5 ± 254.1 (277.5)				
**Control**	354.2 ± 255.9 (436.5)	0.521	35.6	-89.7	161.09
Data presented as Mean ± SD (IQR). Mann-Whitney test. IL-33: Interleukin 33, CI: Confidence interval					

**Table 2:** The observed serologic patterns and the mean differences of CRP, IgG, IgM, and IgA between study groups


					**95% CI**	
**Variables**	**Study group**	**Mean ± SD (IQR)**	**P-value**	**Mean difference**	**Lower**	**Upper**
						Case	3.21 ± 0.81 (1)				
**CRP (mg/l)**	Control	2.90 ± 1.04 (2)	0.277	-0.30	-0.76	0.15
		Case					18.47 ± 9.0 (18.66)				
**IgG (mg/l)**	Control	17.34 ± 8.3 (13.95)	0.162	-1.13	-5.4	3.1
		Case					1.89 ± 0.92 (1.30)				
**IgM (mg/l)**	Control	1.25 ± 0.62 (0.65)	0.003	-0.64	-1.03	-0.25
		Case					3.24 ± 1.68 (2.60)				
**IgA (mg/l)**	Control	3.05 ± 1.69 (2.95)	0.468	-0.18	-1.01	0.64
	Mann-Whitney test. CRP: C-reactive protein, IgG: Immunoglobulin G, IgM: Immunoglobulin M, IgA: Immunoglobulin A, CI: Confidence interval, IQR: Interquartile range						

## 4. Discussion

This study compared IL-33, CRP, IgM, IgG, and IgA in RPL and control group. As a result of this study, only IgM levels showed astatistically significant difference between the case and control groups. Recent research has shown that IL-33 is expressed in endothelial and smooth muscle cells in the placenta, chorioamniotic membranes, and umbilical cord. Also, some studies have shown that polymorphism of IL-33 was correlated with RPL risk comparing control group, and it gave further details that rs1929992 (IL-33) polymorphisms could be associated with RPL in the Iranian population (13). Yue notes that IL-33 serum levels are significantly lower in idiopathic recurrent miscarriage cases than in the control group. Activation of the IL-33 suppression of tumorigenicity 2 signaling pathway in human endometrial stromal cells has been reported to be critical for a successful pregnancy. At 6 wk gestation, a significant increase in IL-33 serum levels were observed in patients who are to be at risk for abortions compared to healthy control pregnancies (14). It seems that when interpreting IL-33 levels, consideration of the week of pregnancy is essential and should be considered. On the other hand, the larger sample size and differences in geographic area in Yue's study should be noted in justifying the difference between the results of our study and the aforementioned study.

Finally, it can be summarized that the IL-33 serum level, at least 4–6 months after the last abortion (in the case group) and the previous live delivery (in the control group), did not show a significant difference between the 2 groups. However, due to the different results of earlier studies in this field, there is still a need for further studies. The results of the present study showed no significant difference in the IL-33 serum level between normal women without recurrent miscarriages and women with a history of recurrent miscarriages. However, the mean IL-33 serum level was slightly lower in women with a history of recurrent miscarriage, which could be a significant difference in larger samples due to the limited sample size in this study.

A protein phenotype may be related to the ethnicity and racial status of the patient; hence more extensive studies in this field are essential. Other suggestions include repetition of studies similar to the follow-up period of most patients to identify the IL-33 serum level more accurately in different conditions and times, designing new studies to identify pathways affecting changes in IL-33 serum levels outside of pregnancy. Moreover, determination and comparison of changes in IL-33 serum levels up to the 20 ${\;}^{\text{th}}$ wk of pregnancy, determining the cytokine phenotype in patients according to their demographic conditions, determining the genotype of this cytokine in patients according to their demographic conditions, they could be suitable research fields in the future.

## 5. Conclusion

In conclusion, in this study, IgM was found to be significantly increased in patients with recurrent miscarriage. This relationship was not observed in IL-33 and other immunological factors. Therefore, the role of IgM as an acute inflammatory marker in recurrent miscarriage has been confirmed. It seems that different aspects of the effects of IL-33 as a predictive biomarker in PRL, should be evaluated in more comprehensive studies.

##  Data availability

Data supporting the findings of this study are available upon reasonable request from the corresponding author.

##  Author contributions

Hossein Ali Khazaei and Farahnaz Farzaneh designed the study and conducted the research. Saeedeh Sarhadi, Javid Dehghan Haghighi, Forough Forghani, Vahid Sheikhi, Bahman Khazaei, and Lida Asadollahi, monitored, evaluated, and analyzed the results of the study. Further Saeedeh Sarhadi and Hossein Ali Khazaei reviewed the article. All authors approved the final manuscript and take responsibility for the integrity of the data.

##  Conflict of Interest

All authors declare that there is no conflict of interest.
